# A New Sebecid from the Paleogene of Brazil and the Crocodyliform Radiation after the K–Pg Boundary

**DOI:** 10.1371/journal.pone.0081386

**Published:** 2014-01-15

**Authors:** Alexander W. A. Kellner, André E. P. Pinheiro, Diogenes A. Campos

**Affiliations:** 1 Laboratório de Sistemática e Tafonomia de Vertebrados Fósseis - Departamento de Geologia e Paleontologia, Museu Nacional - Universidade Federal do Rio de Janeiro, Rio de Janeiro, Brazil; 2 Laboratório de Macrofósseis - Departamento de Geologia, Universidade Federal do Rio de Janeiro, Rio de Janeiro, Brazil; 3 Museu de Ciências da Terra, Serviço Geológico do Brasil - Companhia de Pesquisa de Recursos Minerais, Rio de Janeiro, Brazil; University of Pennsylvania, United States of America

## Abstract

A new crocodyliform, *Sahitisuchus fluminensis* gen. et sp. nov., is described based on a complete skull, lower jaw and anterior cervical vertebrae collected in the São José de Itaboraí Basin of Rio de Janeiro, Brazil. The specimen is one of the best preserved crocodyliforms from Paleocene deposits recovered so far and represents a sebecosuchian, one of the few clades that survived the Cretaceous-Paleogene biotic crisis. The new taxon is found in the same deposit as an alligatoroid, a group that experienced large diversification in the Paleogene. The sebecosuchian record suggests that after the Cretaceous-Paleogene biotic crisis, the less specialized members of this clade characterized by a higher number of teeth compared to the baurusuchid sebecosuchians survived, some having terrestrial habits while others developed a semi-aquatic life style (e.g., *Lorosuchus*). Starting in the Eocene, sebecid sebecosuchians became specialized with a more accentuated oreinirostry as observed in *Sebecus* and in *Langstonia*, but not showing the typical reduced dentition developed by the Cretaceous baurusuchid sebecosuchians. The basal position of *Barinasuchus arveloi*, a high-snouted Miocene sebecid, indicates the occurrence of an independent lineage sometime after the K-Pg biotic crisis that developed accentuated oreinirostry, suggesting a more complex history of the post-K-Pg crocodyliform radiation.

## Introduction

Currently crocodyliforms are worldwide distributed in tropical and subtropical regions in relative low numbers and diversity, consisting of 24 to 30 species (e.g. [1]). All are considered semiaquatic ambushers but their fossil record reveals a much richer evolutionary history in terms of anatomy and ecomorphospaces [2], [3]. Particularly during the Cretaceous, the diversity of those reptiles was much higher and they occupied several distinct niches.

As a natural question, researchers tried to understand the crocodyliform decrease in diversity after the Cretaceous-Paleocene (K-Pg) extinction crisis but this discussion is hampered by the scarce nature of their remains in Paleocene deposits (e.g. [4]). Besides the marine dyrosaurids that have survived the K-Pg boundary and diversified during the Paleocene (e.g. [5], [6]), there are only a limited number of Paleocene specimens described so far, most of which are fragmentary and poorly preserved (e.g. [4], [7]–[9]). This contrasts with the high abundance of Late Cretaceous crocodyliforms, particularly in Brazil, which is even higher than in other Gondwanan areas.

**Figure 1:**
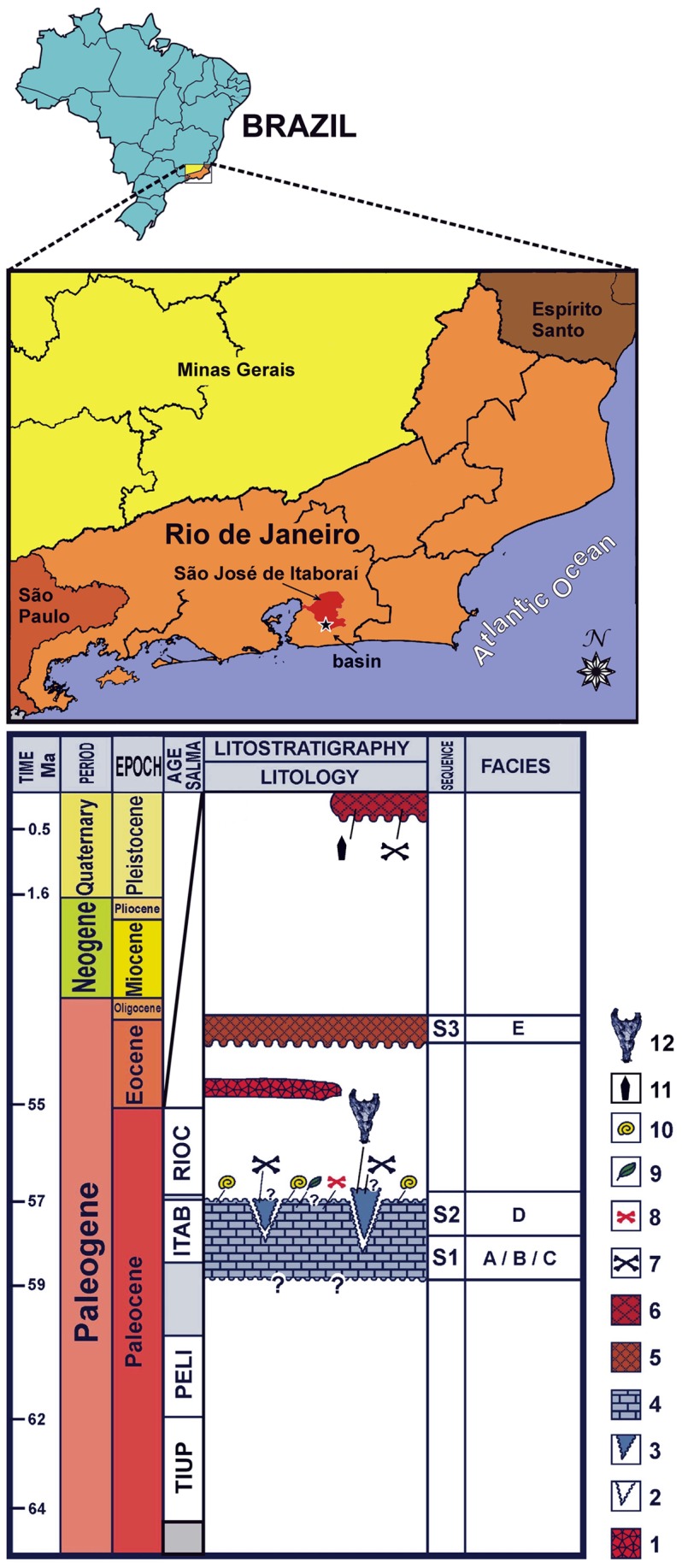
Itaboraí Basin location (A); (B) Itaboraí Basin lithochronoestratigraphic column. 1, ankaramite layer; 2, karst channels and fissures; 3, infilling fissures (Sequence S2, Facies D); 4, main calcareous deposit (Sequence S1, Facies A, B and C); 5, alluvial deposits (Sequence S3, Facies E); 6, clastic sediments; 7, abundant vertebrate fossils; 8, rare vertebrate fossils; 9, plant remains; 10, terrestrial gastropod fossils; 11, archaeological artifacts; 12, *Sahitysuchus fluminensis* gen. et sp. nov. (MCT 1730-R). Stratigraphic column modified after [12], [21].

During the exploration of the São José de Itaboraí Basin (Rio de Janeiro State, Southeast Brazil) that lasted for about five decades and ended in 1984 [10], hundreds of fossil vertebrates were collected (Figure 1). The vast majority is housed at the Earth Science Museum (now at the Companhia de Pesquisa de Recursos Minerais – CPRM), in Rio de Janeiro, and consists of fragmentary remains representing mainly mammals (e.g. [11], [12]). However, some reptiles have also been collected, including the remains of crocodyliforms [13], [14], with only one species formally proposed so far [15].

Among the few well preserved and more complete crocodyliform material is an almost complete skull and lower jaw (MCT 1730-R) that was briefly mentioned (but never figured) in the literature [8], [13], [16] and remained undescribed until now. This specimen represents a new taxon, *Sahitisuchus fluminensis* gen. et sp. nov., and shows that during the Paleocene the São José de Itaboraí crocodyliform fauna was composed by rather primitive (i.e., Sebecosuchia) and more derived (Alligatoridae) post-K-Pg taxa. Such a combination of sebecosuchians and eusuchians has not been previously reported in any deposit so far.

## Materials and Methods

### Phylogenetic Analysis

In order to access the phylogenetic position of *Sahitisuchus fluminensis* gen. et sp. nov., a phylogenetic analysis was performed using the data matrix published by Pol et al. (2012) [17]. Regarding *Sebecus*, we have followed Paollilo & Linares [9], who have restricted this genus to the type species (*Sebecus icaerohinus*) and regarded *“S”. huilensis* as belonging to the genus *Langstonia*. A total of 89 crocodyliform taxa including the new species and 347 characters were used. Parsimony analyses using TNT [18] with heuristics search strategy (10.000 replicates of Wagner trees, 15.000 max. tree in memory) by TBR algorithm were performed. The analyses were run using unordered and ordered characters (1, 3, 6, 10, 23, 37, 43, 44, 45, 49, 65, 67, 69, 73, 77, 79, 86, 90, 91, 96, 97, 104, 105, 106, 108, 116, 126, 140, 142, 143, 149, 167, 176, 182, 197, 226, and 339). Information for *Sahitisuchus fluminensis* gen. et sp. nov. used in the data matrix [17] is as follows:

10[0/1]?????12 ???0??-000 11011[0/1][0/1]00? 0100022110 100011?11? 1101010?10

?0103?[1/2]12- [2/3]10?101?21 ????????1? ?0???????? ??[1/2]01?00?? ?1?????[0/1]10

01??????0? ??0??00?01 0[0/1]2?1??001 010[0/1]11?1?0 ?1?0?0110- 1?01???110

[0/1]?100000?0 0101?10010 0001?[0/1]0001 01?1000010 ???00????? [0/1]0010[0/1]????

??0?0?0000 0100?0?0?? ?10?00?01? -???00?010 0??????[0/1]00 0001010??? ??????????

?????????? ?????????? ?????????? ???????

For more information see Supporting Information (Data S1, Figures S1 and S2).

### Nomenclatural Acts

The electronic edition of this article conforms to the requirements of the amended International Code of Zoological Nomenclature, and hence the new names contained herein are available under that Code from the electronic edition of this article. This published work and the nomenclatural acts it contains have been registered in ZooBank, the online registration system for the ICZN. The ZooBank LSIDs (Life Science Identifiers) can be resolved and the associated information viewed through any standard web browser by appending the LSID to the prefix “http://zoobank.org/”. The LSID for this publication is: urn:lsid:zoobank.org:pub: 322EE489-D9D2-4CE6-9DAF-36E30C03881D. The electronic edition of this work was published in a journal with an ISSN, and has been archived and is available from the following digital repositories: PubMed Central, LOCKSS.

No permits were required for the described study, which complied with all relevant regulations. See appropriate section of Systematic Paleontology for locality, stratigraphic, repository and specimen number.

## Results

### Systematic Paleontology

**Mesoeucrocodylia** Whetstone *&* Whybrow, 1983 [19], *sensu* Benton & Clark, 1988 [20]

**Sebecosuchia** Simpson, 1937 [7]

**Sebecidae** Simpson, 1937 [7]

***Sahitisuchus fluminensis*** **gen. et sp. nov.**

urn:lsid:zoobank.org:act:10A04487-436F-4509-BFDD-42B4DF6B8177

#### Derivation of name

Generic name *Sahiti* comes from the Xavante culture (sahi ti), one of the indigenous Brazilian inhabitants, meaning “to be angry” or “to be brave”, in allusion to warriors; and *souchos*, refers to the Egyptian crocodile god. Specific name *fluminensis* is a latinization of fluminense, designation of citizens born in the Rio de Janeiro State.

#### Type species

Almost complete skull and lower jaw, proatlas, intercentrum, the axis and the 3^rd^ cervical vertebra (MCT 1730-R), housed at the Museu de Ciências da Terra, Companhia de Pesquisas de Recursos Minerais (CPRM), Rio de Janeiro, Brazil (cast at the Museu Nacional/UFRJ - MN 4711-V).

#### Type locality, and horizon and age

São José Farm, São José de Itaboraí Municipal District, ENE in the Rio de Janeiro Metropolitan Área (SE Brazil; 22°50′20″S and 42°52′30″W). Collected in the S2 sequence [21]; Itaboraian SALMA (South American Land Mammals Age), middle Upper Paleocene, 58,2-56,5 Ma [22].

#### Diagnosis

Sebecid crocodyliform with the following autapomorphies: mandible lacking external mandibular fenestra; and odontoid process fused to the axis with vertical anterior surface that lacks medial processes. The new species can be further distinguished from other sebecids by the following combination of characters: infraorbital jugal region with shallow ventrolateral depression (shared with *Lorosuchus*); shallow elliptical depression on the posterior surface of the quadrate close to the craniomandibular articulation (shared with *Sebecus icaeorhinus*); rough and rugose dorsal edge of supratemporal fossa (shared with *Sebecus icaeorhinus*); sharp, semilunate exoccipital posterior processes, directed medially (shared with *Ayllusuchus*); jugal posterior process higher than anterior process and lateral expanded (shared with *Bretesuchus*); rough longitudinal ridge on the lateroventral edge of angular and dentary, ending close to the mandibular symphysis level (shared with *Bretesuchus*, *Sebecus*).

### Description and Comparisons

**Figure 2:**
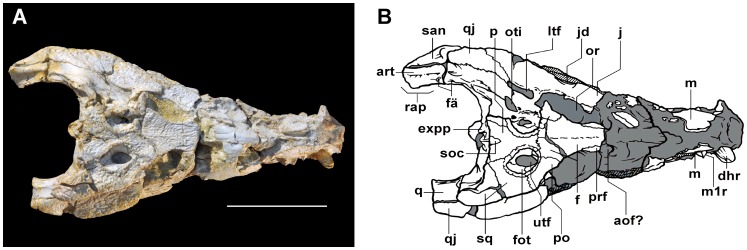
*Sahitysuchus fluminensis* gen. et sp. nov. (MCT 1730-R), in dorsal view. A, photo; B, illustration. **9–12th mlt**, eighth to eleven left maxillary teeth; **an**, angular; **anr**, angular ridge; **afo**, mandibular adductor fossa; **aof?**, antorbital fenestra?; **art**, articular; **bs**, basisphenoid; **bo**, basioccipital; **boc**, basioccipital middle crest; **bot**, basioccipital basal tubera; **chg**, choanal groove; **cq**, cranio-quadrate passage; **d**, dentary; **dhl**, left hypertrophied replacement tooth; **dhr**, right hypertrophied dentary tooth; **ec**, ectopterygoid; **expp**, exoccipital posterior process; **f**, frontal; **fä**, foramen aereum; **fcp**, foramen caroticum posterius; **fic**, foramen intermandibularis caudalis; **fme**, median Eustachian foramen; **fot**, upper temporal fossa; **fra**, fractured area; **fv**, foramen vagi; **int**, intercentrum; **j**, jugal; **jd**, jugal latero-ventral depression; **l**; lachrymal; **lptyp**, lateral pterygoidal process (flange); **ltf**, laterotemporal fenestra; **m**, maxilla; **m1l**, first left maxillary tooth; **m1r**, first right maxillary tooth; **m3l**, third left maxillary tooth; **m3r**, third right maxillary tooth; **m4l**, fourth left maxillary tooth; **m5l**, fifth left maxillary tooth ; **nc**, nuchal crest; **occ**, occipital condyle; **or**, orbit; **oti**, otic incisure; **oto**, otoccipital; **p**, parietal; **pl**, palatine; **pmt**, posterior maxillary teeth; **po**, postorbital; **pop**, para-occipital process; **pfr**, prefrontal; **pro**, proatlas; **pty**, pterygoid; **q**, quadrate; **qd**, quadrate depression; **qdc**, quadrate dorsal crest; **qj**, quadratojugal; **rap**, retroarticular process; **san**, surangular; **sanr**, surangular lateral ridge; **sf**, siphoneal foramen; **soc**, supraoccipital; **sof**, suborbital fenestra; **spl**, splenial; **sq**, squamosal; **sqp**, squamosal posterior process (squamosal prong); **utf**, upper temporal fenestra; **XII**, twelfth cranial nerve exit. Scale bar: 100 mm.

**Figure 3:**
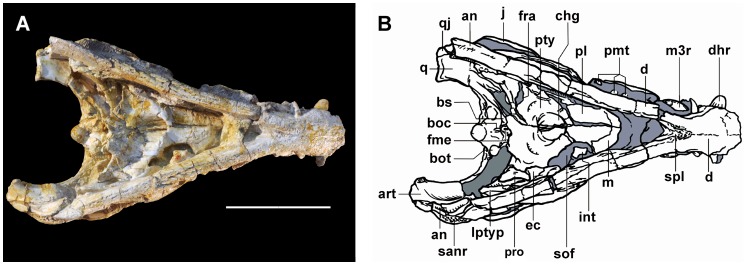
*Sahitisuchus fluminensis* gen. et sp. nov. (MCT 1730-R), in ventral view. A, photo; B, illustration. For abbreviations see Figure 1. Scale bar: 100 mm.

**Figure 4:**
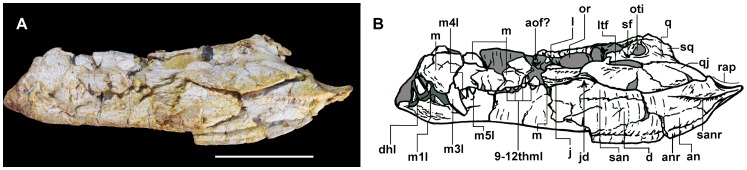
*Sahitisuchus fluminensis* gen. et sp. nov. (MCT 1730-R), in left lateral view. A, photo; B, illustration. For abbreviations see Figure 1. Scale bar: 100 mm.

**Figure 5:**
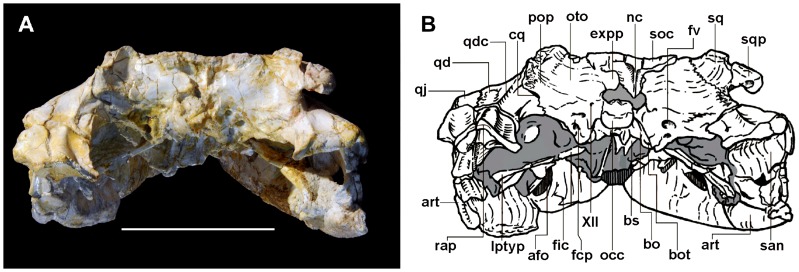
*Sahitisuchus fluminensis* gen. et sp. nov. (MCT 1730-R), in occipital view. A, photo; B, illustration. For abbreviations see Figure 1. Scale bar: 100 mm.

**Figure 6:**
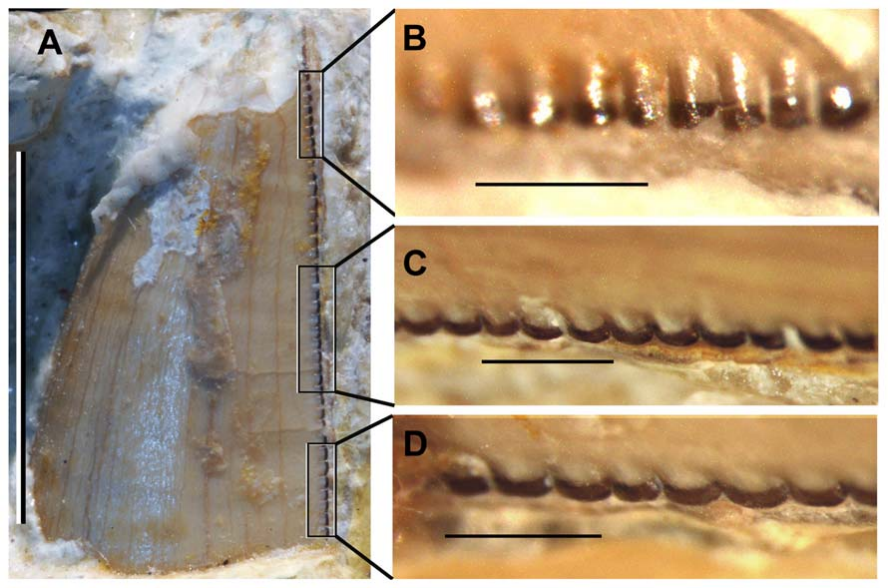
Fourth mandibular tooth from the left side of *Sahitisuchus fluminensis* gen. et sp. nov. (MCT 1730-R), showing the serrations. A, labial surface; B, detail for apex carina; C, detail for middle carina; D, detail for basal carina. Scale bar in A: 10 mm; B, C and D: 1 mm.

**Figure 7:**
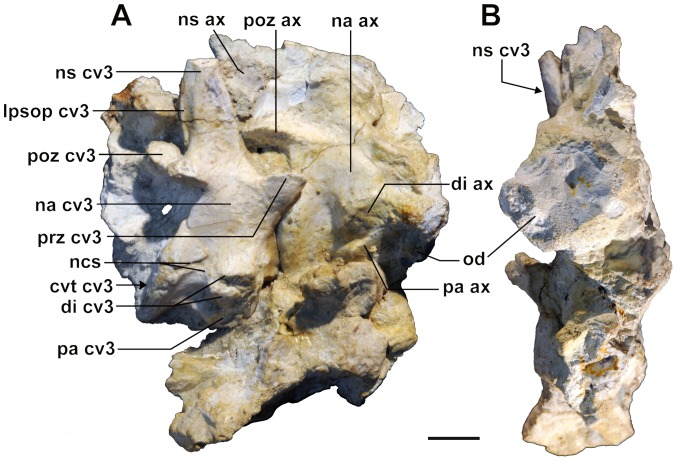
*Sahitisuchus fluminensis* gen. et sp. nov. (MCT 1730-R) cervical vertebrae. A, right lateral view of axis and third cervical vertebra; B, anterior view, showing the odontoid process. **cvt cv3**, centrum of third cervical vertebra; **di ax**, axis diapophysis; **di cv3**, diapophysis of third cervical vertebra; **lpsop cv3**, postspinal lamina of third cervical vertebra; **na ax**, axis neural arch; **na cv3**, neural arch of third cervical vertebra; **ncs**, neuro-central suture; **ns ax**, axis neural spine; **ns cv3**, neural spine of third cervical vertebra; **od**, odontoid process; **pa ax**, axis parapophysis; **pa cv3**, parapophysis of third cervical vertebra; **poz ax**, axis postzygapophysis; **poz cv3**, postzygapophysis of third cervical vertebra; **prz cv3**, prezygapophysis of third cervical vertebra. Scale bar: 10 mm.

Overall the material of *Sahitisuchus fluminensis* is well preserved consisting of the skull, lower jaw and cervical elements (Figures 2–7; Tables 1, 2). Although some dorsoventral crushing is observable it was not severe to affect the shape of most cranial elements, including the rostral end that kept most of original anatomy. The most affected area was the more posterior portion of the skull, with some elements, particularly the supraoccipital displaced towards the foramen magnum.

**Table 1:** Measurements in mm of *Sahitisuchus fluminensis* gen. et sp. nov. (MCT 1730-R), adapted from [25] ∼, estimated measurement.

**1**. greatest width	*179*
**2**. width of rostrum, posterior	*97*
**3**. interorbital distance	*60*
**4**. orbit length	*52.2*
**5**. skull table width, anterior	*67*
**6**. skull table length	*96*
**7**. skull table width, posterior	∼*143.8*
**8**. occipital condyle width	*23*
**9**. occipital condyle height	*15*
**10**. orbit width	*26.6*
**11**. choana width	*39*
**12**. choana length	*35.5*
**13**. skull roof length	*67*
**14**. quadrate condyle width	*37.7*
**15**. supratemporal fossa width	*28.4*
**16**. supratemporal fossa length	*34.4*
**17**. palatal fenestra length	∼*48.6*
**18**. pterygoid flanges width	∼*125.5*
**19**. rostrum width at secondary dental peak	∼*50.3*
**20**. rostrum width at notch (or fossa) for 4^th^ mandibular tooth	*∼35.4*
**21**. palatine bar width	*38*
**22**. mandible length	∼*369*
**23**. symphysis length	∼*84.2*
**24**. retroarticular process length	*44*
**25**. distance between supratemporal fossa/fenestra	*17*
**26**. distance between medial borders of supratemporal fossae	*15.3*
**27**. supratemporal fenestra width	*13.8*
**28**. supratemporal fenestra length	*22*
**29**. distance between supratemporal fossa and lateral margin of skull roof (at po–sq suture level)	*21*
**30**. distance between supratemporal fossa and posterior margin of skull roof	*22*
**31**. quadrate distal body length	*31.5*
**32**. laterotemporal fenestra length	∼*38.6*
**33**. occiput height (dorsal skull roof surface to occipital condyle)	*38*
**34**. occiput height (dorsal skull roof surface to medial exoccipital ventral margin)	*54.7*

**Table 2:** *Sahitisuchus fluminensis* gen. et sp. nov. (MCT 1730-R) teeth measurements in mm.

Right tooth row			Left tooth row		
tooth	fabl	tch	tooth	fabl	tch
m1	*4.8*	*14.8*	m1	*10.9*	*6*
m2	*5.2*	*9.2*	m2	*14*	*7.5*
m3	*31.5*	*11.7*	m3	*28.7*	*15.8*
m4	*∼11.7*	*∼5.8*	m4	*10*	*6.4*
lmt	*6.4*	*6.1*	m5	*8.2*	*5.7*
1^st^d (d4 ?)	*∼26.2*	*∼15.6*	6^th^m	*–*	*9.3*
2^nd^d	*14.8*	*7.7*	7^th^m	*6.14*	*6*
3^th^d	*8.6*	*5*	8^th^m	*6.5*	*7.2*
4^th^d	*–*	*6.6*	9^th^m	*–*	*5.7*
			10^th^m	*6.4*	*6.1*
			1^st^d (d4 ?) s	*∼12.8*	*7.1*

**1std (d4 ?)**, first exposed dentary tooth of the right side; **1std (d4 ?) s**, first exposed replacement dentary tooth of the left side; **d**, dentary tooth; **fabl**, fore-after basal length; **lmt**, last exposed maxillary tooth; **m**, maxillary tooth; **tch**- tooth crown height; –, lack information; ∼, estimated measurement.

The premaxillae and the region of the external nares were broken off and the skull was slightly compressed dorsoventrally during preservation. The lower jaw is occluded with the skull and some bones lack the external bone cortex. In dorsal view, the skull of *Sahitisuchus fluminensis* is elongated, showing two slight constrictions at about the level of the 5th and 11th maxillary alveoli (Figures 2A, B). It differs from *Sebecus icaeorhinus* and *Stolokrosuchus* by being comparatively shorter [23], [24], but not as short as *Lorosuchus* [4]. *Sahitisuchus* does not show the same oreinirostral condition as *Sebecus*, *Barinasuchus*, *Bretesuchus*, *Zulmasuchus* and *Langstonia* [8], [9], [16], [23], [25].

### Cranial bones

The skull-roof is flat and rectangular, being wider than long. The **supratemporal fossa** is much larger than the supratemporal fenestra (Figures 2A, B). This fossa is about three times smaller than the orbits. The distance between the supratemporal fossae is half that of the frontal inter-orbital width. The dorsal border of this fossa is surrounded by rugosities, forming an elevation that is more developed in the medial and lateral borders. This condition is similar to *Sebecus* and the peirosaurid *Hamadasuchus*, differing from any other mesoeucrocodyliforms (*sensu* Benton & Clark 1988 [20]) known to date. The **palpebral** bones, which are present as anterior and posterior elements in other sebecids (e.g. *Sebecus*, Lumbrera form [4], [26]) and specially in “peirosaurids” (e.g. *Lomasuchus*, *Uberabasuchus*, *Montealtosuchus*) [27]–[29], are not preserved. Even so, the orbit is placed rather laterally, a typical terrestrial sebecid feature, and not laterodorsally as in semi-aquatic crocodyliform morphotypes (e.g., *Stolokrosuchus*, *Lorosuchus* [4], [24], and extant species). The **frontal** is broad and triangular, with a low and smooth longitudinal crest running from the middle part to the posterior portion this bone. A longitudinal frontal crest is a common characteristic for many basal mesoeucrocodylian species, which includes some sebecosuchian taxa (e.g. *Sebecus*; *Zulmasuchus*; *Iberosuchus macrodon*; *Pepesuchus*, *Lorosuchus* [4], [16], [23], [30]. The frontal ornamentation is similar to that on the maxilla, with the wrinkles starting at the frontal longitudinal crest directed to the lateral margins. The **jugal** is very large and ornamented like most other cranial bones. The posterior ramus is laterally expanded and higher than the anterior one, an unusual feature within sebecosuchians only previously observed in *Bretesuchus* [8]. The ventrolateral margin is concave, a unique feature among basal mesoeucrocodylians. The **quadratojugal** takes part in the cranio-mandibular articulation forming the “double articulation” (*sensu* Buffetaut 1975 [31]), a feature absent in extant eusuchians but observed in all sebecid species and some other not closely related taxa (e.g. *Trematochampsa*, *Libysocushus*, Dyrosauridae). The **squamosal** shows a developed sculptured dorsal posteriorly pointed lobe (the squamosal posterior process or the squamosal prong [32] that is directed posteriorly and does not form a horn, similar to *Hamadasuchus* and *Lomasuchus* [27], [32]. *Sebecus* also shows such a developed process, but differs from *Sahitisuchus* by a more squared-shape posterior end [23]. The **quadrates** are massive and mostly unsculptured. The portion of the tympanic cavity formed by the quadrate is not multifenestrated like the one found in protosuchians, notosuchians [33] and baurusuchids (e.g. [34]). It also lacks the oblong concavity, which is characteristic of the Baurusuchidae [34]–[36] but, like *Sebecus*, *Hamadasuchus* and recent species, shows only two openings: the small, anterior preotic siphonial foramen, followed by the larger, oval otic incisure. The ventral portion of the tympanic membrane was supported by a low and sharp semicircular crest. The quadrate distal body extends beyond the occipital limits and bears a well-developed sharp crest that runs from the lateral region of the cranioquadrate passage to the end of this bone. A semi-elliptical shallow concavity in the most distal portion of the quadrate body, just medial to the quadratojugal-quadrate suture and anterior to the cranial-mandibular articulation is very conspicuous in the new species (Figures 2, 5). In ventral view the quadrate exhibits pronounced crests A and A′ [37] for *M. adductor mandibulae posterior* [38].

### Palatal region

The anterior process of the **palatine** projects over the maxillary palatal shelf with a “U-shaped” anterior margin [39], which extends well forward from the anterior margin of the suborbital fenestrae (Figure 3). The **pterygoids** are broad wing-shaped elements, similar to those found in *Sebecus* (MMP 235), being distinct from the broad quadrangular pterygoid of *Zulmasuchus*, peirosaurids (e.g. *Montealtosuchus*, *Hamadasuchus*) and derived eusuchians. The lateral border of the pterygoid flanges are arched and curved inwards similar to *Zulmasuchus* [9], [16]. In *Bretesuchus* this curvature is even more accentuated than in the latter species [8]. The pterygoid plate is slightly concave, very large and broad. The **basicranium** is not verticalized as found in Eusuchia but more verticalized than some basal crocodyliforms forms (e.g. baurusuchids and sphagesaurids) (Figures 3, 5). The **choanae** are positioned between the palatine and pterygoid, having a low and laminar choanal septum. The choanal groove (or fossa) is circular as the one of *Sebecus* and *Barinasuchus* but comparatively smaller than in these taxa.

### Occipital region

The occiput is about four times wider than high, which is partially attributed to crushing of the specimen (Figure 5). On the skull roof, the **supraoccipital**, this bone is as a relatively small forward pointed triangle which is inserted between the parietals. In occipital view this bone is relatively large and exhibits a prominent nuchal crest. The latter comprises the insertion point for *M. spinalis capitis* [40]. The posttemporal fenestrae are not well preserved due to compression, but the preserved part is very reduced with no postoccipital process. The supraoccipital descending portion is acute and reaches the foramen magnum. However, this seems more the product of the compression than to an autapomorphic feature of the new species. The **exoccipital** has a very distinct mediodorsal process similar to *Ayllusuchus*. This process is sharp and has a semilunate shape, comprising the insertion point for the *M. rectus capitis sublimus* and *M. spinalis capitis*. The **basioccipital** is trapezoidal and positioned oblique (∼45°) relative to the horizontal plane. This bone possesses a median elevated crest (insertion point for the *M. rectus capitis anterior*). The **basisphenoid** is short and completely verticalized, being little exposed both in occipital and palatal views. Similar inclination of basisphenoid and basioccipital is also found in some sebecids (e.g. *Zulmasuchus* and *Bretesuchus*) and peirosaurids.

### Mandible

In ventral view the mandible shows an inverted “Y-shape” (Figures 3, 4). The robust mandibular symphysis is formed by the dentaries and splenials and reaches to opposite the fourth maxillary teeth while the dentary teeth are not visible, occupying about 21% of the mandibular length. In lateral view the anterior mandible portion is not as high as those of *Bretesuchus* and baurusuchids. The **splenial** forms about one-fourth of the mandibular symphysis and medially covers the Meckelian channel as a vertical and thick bone lamina. The **mandibular lateral fenestrae** is closed, an unusual feature in crocodyliforms and differing from all other sebecosuchian taxa. The **angular** exhibits a robust well-developed ventrolateral ridge that runs over almost the entire angular length, probably corresponding to the insertion area for the strongest component of the mandibular adductory musculature (*M. adductor mandibulae internus pterygoideus ventralis* [38]). The **surangular** takes part in the glenoid fossa, is stout and slightly ornamented. In lateral view, the suture with the dentary is gently convex. The dorsal margin of this bone is arched. A developed ridge with a smooth dorsal and rugose ventral surface is present below the glenoid fossa (Figure 3A, B). Except for *Bretesuchus*, in all other sebecosuchids where this region is preserved this crest is only incipient or poorly developed. With the retroarticular process, this structure is regarded as the probable insertion point for the components of the *M. depressor mandibulae* component [38], [41]. The **articular** forms about 60% of the glenoid fossa, like in other sebecids but unlike some other sebecosuchians, as in baurusuchids. The retroarticular process shows an elevated lateromedially crest just posterior to the glenoid fossa, a structure that does not allow palinal-propalinal jaw movements. The **retroarticular process**, formed by the articular and the surangular, is arched with a concave dorsal margin. The most medial posterior part, formed by the articular, is “tongue shaped”. Seen from posterior view, the posterior margin is inclined ventromedially forming an angle of about 40° relative the horizontal plane. A blunt crest runs longitudinally in the articular portion of this process. The retroarticular foramen aëreum is small and opens close to the medial margin of the retroarticular process, right after the glenoid fossa.

### Dentition

The premaxillary teeth are not preserved but at least twelve maxillary teeth must have been present (Figures 4, 6). Due to the fact that the upper and lower jaws are occluded, most of the mandibular teeth are not visible.

The new species has the crocodyliform plesiomorphic pattern of occlusion [3], with the hypertrophied dentary caniniform exposed laterally, occluding in the premaxillary-maxillary fossa, and the following maxillary teeth occluding buccally relative to the mandibular tooth row. The upper dentition is heterodont with three morphological arrangements, all showing serrated carinae formed by true denticles (*sensu* Langston 1975 [42]) (Figure 6). The first three maxillary teeth are ziphodont, curved posteriorly with pointed and buccolingually compressed crowns. The second dental morphotype is formed by the following two (perhaps three) teeth that are lanceolate and bear straight (i.e., not posteriorly curved) crowns. They are followed by progressively shorter teeth with blunt apices and a marked constriction between root and crown. The large caniform tooth shows about 3–3,5 denticles per mm (Figure 6).

### Cervical elements

Several of the most anterior cervical elements such as the pro-atlas, intercentrum and odontoid process are described for first time in Sebecidae (Figures 3, 7). The **pro-atlas** was displaced over the left pterygoid flange. It is a small and laminar V-shaped bone with a low dorsal crest. Compared to modern crocodilians, this bone is rather conservative differing mainly by being narrower (Figure 3). The **intercentrum**, the only part identifiable of the **atlas**, was also displaced, being preserved over the left suborbital fenestra. This bone is robust, not laminar, with two blunt posteriorly directed processes for the first cervical ribs. The **axis** is preserved associated with a well-developed **odontoid process** that is similar to the one found in recent taxa by being massive and showing on each side blunt anterior tuberous processes (Figure 7). In the new species the anterior region is vertical and lacks the small medial processes found in at least some recent taxa. Furthermore, the odontoid process is fused with the axis with no visible suture indicating that this is most likely a very old individual [43]. The axis is well developed with a low, blade-like neural spine. The **third cervical vertebra** is amphicoelous, with a tall spike-like neural spine, inclined posteriorly, with a postspinal lamina that is bifurcated at the base (Figure 7). As in *Sebecus* (the only other sebecid where this part of the skeleton was described [17]), the diapophysis is divided by the neurocentral suture and in lateral view, the centrum shows a medial constriction and a trapezoidal shape, with anterior and posterior articulations inclined anteriorly. Among the differences with *Sebecus*, *Sahitisuchus* has more robust and broader diapophyses, and the length of the third cervical centrum is subequal compared to the axis.

## Discussion and Conclusions

**Figure 8:**
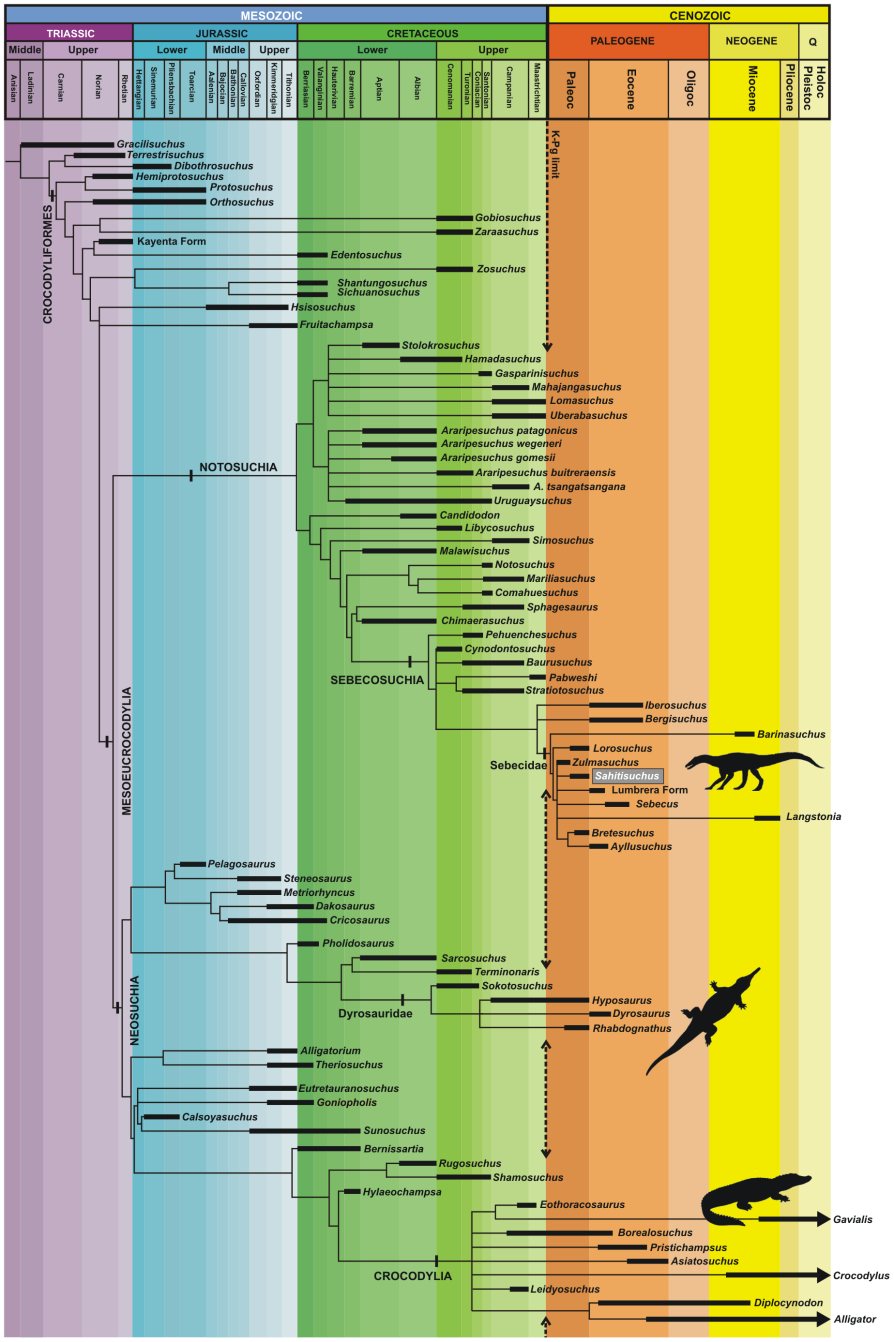
Biochronology of the Crocodylomorpha based on the strict consensus tree obtained by the phylogenetic analysis (see text for details) and recorded temporal range.

In order to investigate the phylogenetic position of *Sahitisuchus fluminensis*, we used the data matrix published by Pol et al. 2012 [17], who have considered all well-known sebecid taxa (see Data S1). The analysis was run through TNT, with characters either unordered or ordered and both results show that *Sahitisuchus* is a member of the Sebecidae (Figure 8). Furthermore, the addition of the new Brazilian taxon collapses the monophyletic genus *Sebecus* recovered in previous studies [17] and suggests that *Barinasuchus* occupies a basal position within the Sebecidae relative to *Lorosuchus*.

The overall crocodyliform record in number of specimens and taxa from Paleocene deposits is rather slim. This low diversity contrasts with the expressive crocodyliform record of the Cretaceous, where a great number of species thrived in a variety of ecological niches, particularly in the Gondwana, such as notosuchians (e.g. Uruguaysuchidae, Sphagesauridae), baurusuchids (e.g. *Baurusuchus*, *Stratiotosuchus*, *Pabweshi*), peirosaurids (e.g. *Uberabasuchus*, *Pepesuchus*, *Hamadasuchus*), mahajangasuchids (e.g. *Mahajangasuchus* and *Kaprosuchus*), and other taxa whose phylogenetic position is more controversial [e.g. 44].

Only three crocodyliform lineages are represented either before and after the K-Pg boundary: the marine Dyrosauridae, particularly abundant in coastal deposits of Africa [45], [46], the semiaquatic Crocodylia, recovered specially by alligatoroids from North and South America [15], [47]–[50]; and the terrestrial sebecosuchians (e.g. [17]). Dyrosaurids are very specialized and became quite diversified after the K-Pg boundary, becoming along with sharks the main marine predators after the demise of mosasaurs [6]. Although the Alligatoroidea were already present in the Late Cretaceous this group only diversified after the K-Pg biotic crises (e.g. [39], [49]), contrary to what happened with the sebecosuchians that became less diverse (with baurusuchids restricted to Upper Cretaceous [e.g. 34]).

So far, the only Paleocene deposit where members of the Alligatoroidea (*Eocaiman itaboraiensis* [15]) and Sebecosuchia (represented by *Sahitisuchus*) were recovered is the São José de Itaboraí Basin. Having its origin related to the separation of South America and Africa, resulting in the opening of the South Atlantic Ocean [51], this tectonic feature consists of a small half-graben with a NE-SW major axis of 1.400 m and a sedimentary sequence that reaches a maximum thickness of 125 m [52]. The main fossils described so far are mammals that record one of the earliest phases of the mammalian radiation in South America after the K-Pg biotic crisis (e.g. [53]). The inferred age of the Itaboraí Basin based on the mammalian fauna has been the matter of a recent debate [21], [53]–[55]. Despite this uncertainty, the new crocodyliform is part of the so-called S2 paleofauna, whose age (Itaboraian SALMA [55]) is considered middle Upper Paleocene varying in absolute terms between 61.8 million to 58.5 million years [12] or 58.5 million to 56.5 million years [22].

The co-occurrence of a remnant of the pre-K-Pg sebecosuchian and a post-K-Pg alligatoroid crocodyliform taxon, here represented by *Sahitisuchus* and *Eocaiman* [15], respectively, in the Paleocene deposits of the Itaboraí Basin is quite unusual and somewhat surprising. The taphonomic history of those specimens, as of other fossils found in this basin, has been difficult to retrieve, particularly due to the fact that all material was recovered from fissures and not detailed information about their collecting has been recorded. This raises the valid question if all fossils were synchronous [e.g.], [ 56–57]. In the lack of other information, some authors have used the color of the specimens to establish if they came from the same or distinct fissures that stands as a proxy for being regarded synchronous [56].

Regarding the crocodyliforms, it is clear that the sebecid and the alligatorid species came from distinct environments (terrestrial and semi-aquatic, respectively) and represent animals that lived around a freshwater lake before becoming preserved. Among the specimens attributed to *Eocaiman*, several show distinct colors suggesting that they come from distinct fissures [15]. The material of *Sahitisuchus fluminensis* is preserved in a greyish colored limestone, similar to some of the *Eocaiman* material. Furthermore, despite the questions about the correct absolute age, it has been proposed that the calcareous deposits of the São José de Itaboraí Basin were formed in a time span of 2 million years [57]–[58] or less. Therefore, we can conclude that *Sahitisuchus* and *Eocaiman* were either set apart for a comparatively short geological timespan or most likely co-occurred, the last hypothesis favored here.

One possible scenario that could explain the co-occurrence of *Sahitisuchus* and *Eocaiman* is that, right after the Cretaceous-Paleogene biotic crisis, only the less specialized crocodyliforms survived (e.g. [59]), except for the marine dyrosaurids that appear not to have been negatively affected by this event (e.g. [6]). Regarding sebecosuchians, this appears to be correct since the Cretaceous forms show high skulls and a marked specialization in the dentition that is quite reduced. The Cretaceous taxa *Baurusuchus* and *Stratiotosuchus*, for example, show only five maxillary teeth opposed to the 10 in the Paleogene *Lorosuchus*, *Bretesuchus* and *Zulmasuchus*, and 12 in *Sahitisuchus*. Furthermore, Paleocene sebecosuchians represented only by the Sebecidae show the posterior teeth blunt and not specialized as in the Cretaceous sebecosuchians. It is conceivable that the Paleocene sebecosuchians adopted a mixture of semi-aquatic and terrestrial lifestyles and therefore might have at least partially shared the same environments as Paleocene alligatoroids. After the Eocene, sebecosuchians became again more specialized, developing a higher and laterally compressed rostrum as observed in the Eocene *Sebecus* and the Upper Miocene *Langstonia*. They further show a trend to reduce dentition (e.g. *Sebecus* exhibiting nine maxillary teeth), although not approaching the reduction observed in the Cretaceous baurusuchid sebecosuchians.

The unexpected result in the phylogenetic study presented here with the addition of *Sahitisuchus* to the data matrix published by Pol et al. [17], is the basal position of the high-snouted *Barinasuchus*. Recovered from Miocene deposits, this very large sebecid is known from the anterior portion of the rostrum only [9]. If its phylogenetic position is correct, this species indicates the presence of a yet another independent sebecid lineage that sometime after the K-Pg biotic crisis developed accentuated oreinirostry (sensu [60]) independently from other sebecids, suggesting a more complex history of the post-K-Pg crocodyliform radiation.

## Supporting Information

Figure S1 **Topology resulted by heuristic analysis of unordered characters states.** Bootstrap values above the lines (branches), at left and no-italic; Jacknife values above lines (branches), at right and italic; Bremer decay below the lines (branches). Data matrix from Pol et al., (2012) [17] with *Sahitisuchus fluminensis* added.
(JPG)

Figure S2 **Topology resulted by heuristic analysis of third seven ordered characters states.** Bootstrap values above the lines (branches), at left and no-italic; Jacknife values above lines (branchs), at right and italic; Bremer decay below the lines (branches). Data matrix from Pol et al., (2012) [17] with *Sahitisuchus fluminensis* added.
(JPG)

Data S1 **Phylogenetic Analyses.**
(DOC)
