# Supplementary figures and images for: A New Sebecid from the Paleogene of Brazil and the Crocodyliform Radiation after the K–Pg Boundary
Source: PLoS One. 2014 Jan 15;9(1):e81386. doi: 10.1371/journal.pone.0081386 (PMC3893294; doi:10.1371/journal.pone.0081386)

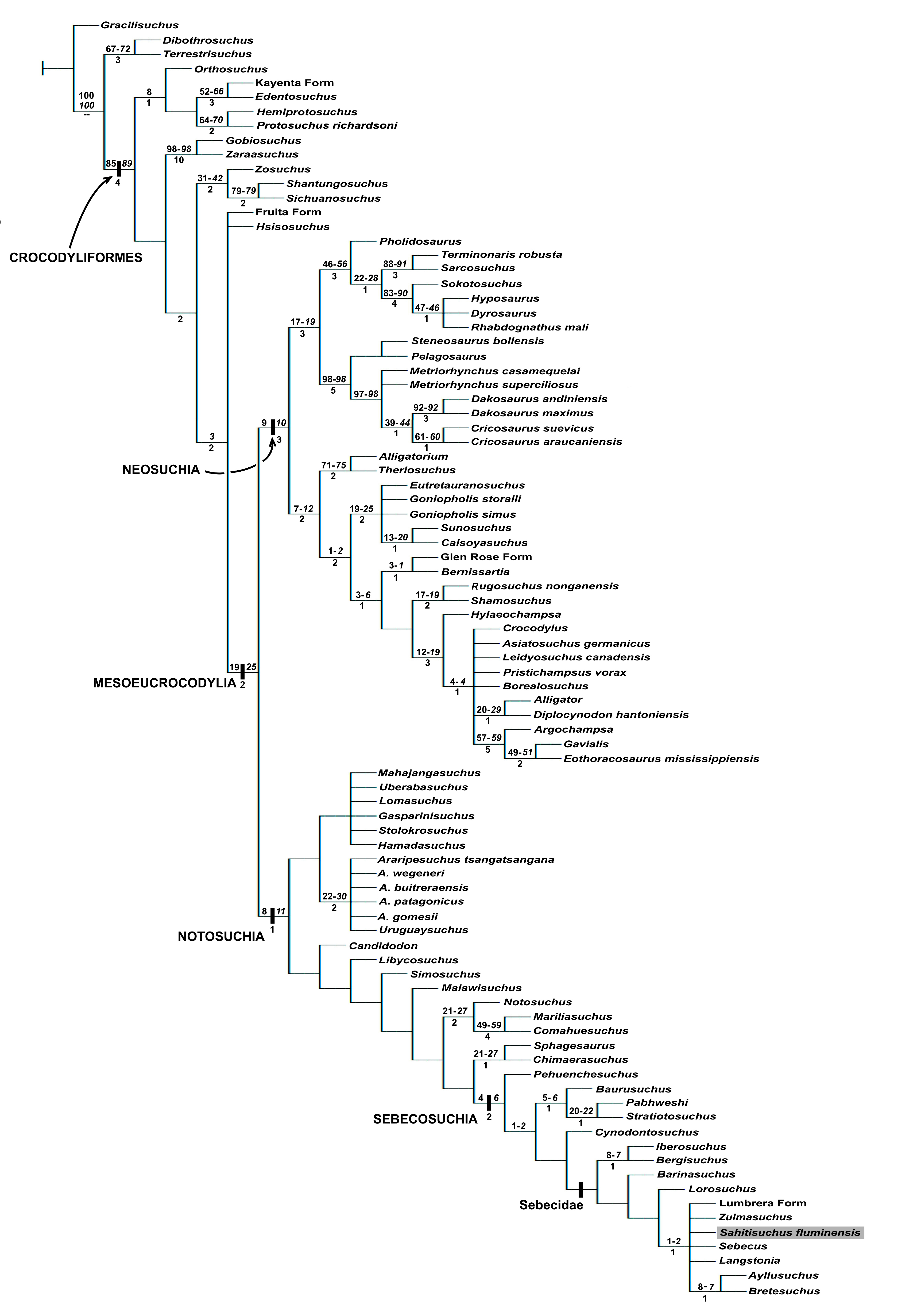

Supplement: Figure S1 — Topology resulted by heuristic analysis of unordered characters states. Bootstrap values above the lines (branches), at left and no-italic; Jacknife values above lines (branches), at right and italic; Bremer decay below the lines (branches). Data matrix from Pol et al., (2012) [17] with Sahitisuchus fluminensis added. (JPG) [file pone.0081386.s001.jpg]

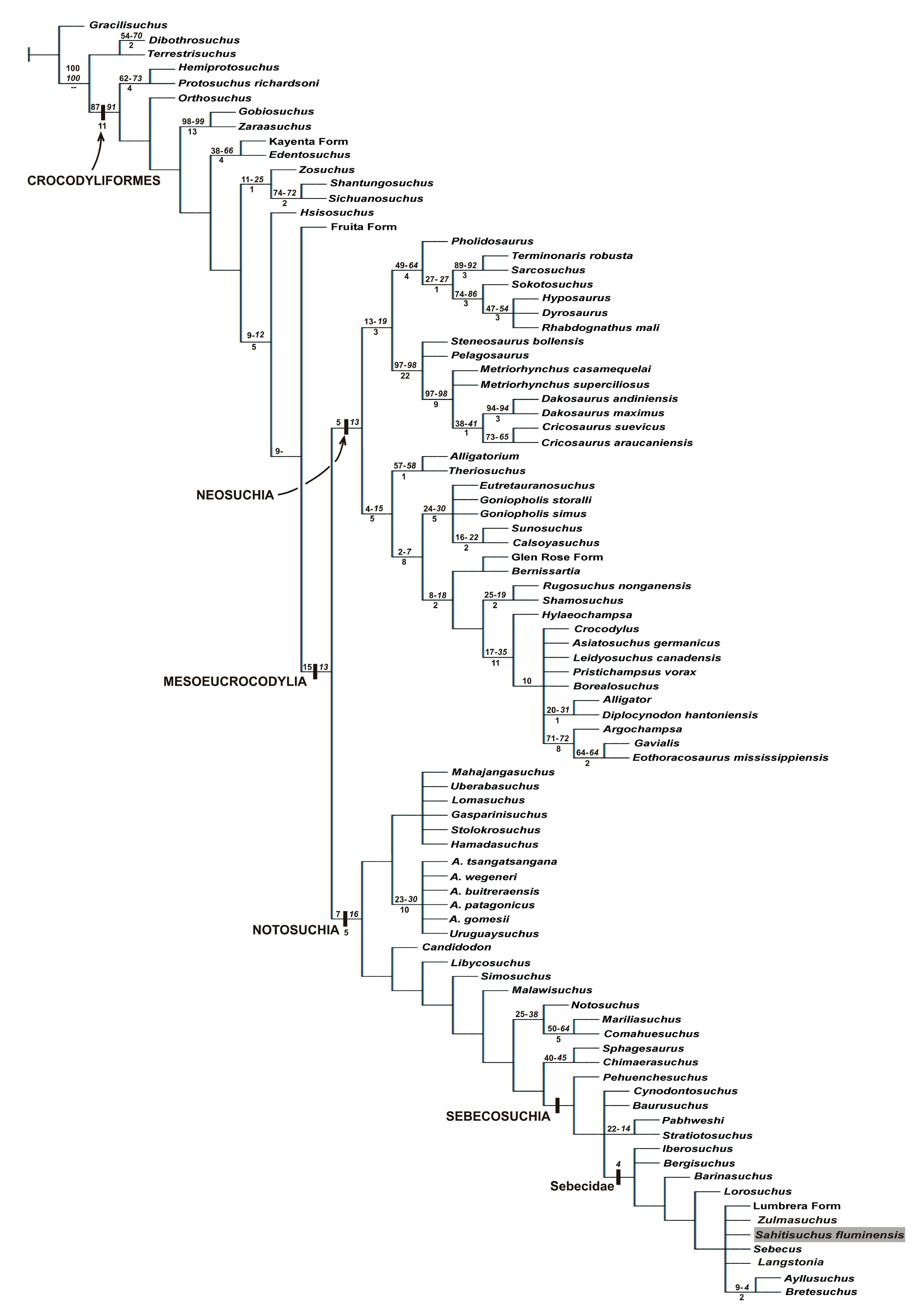

Supplement: Figure S2 — Topology resulted by heuristic analysis of third seven ordered characters states. Bootstrap values above the lines (branches), at left and no-italic; Jacknife values above lines (branchs), at right and italic; Bremer decay below the lines (branches). Data matrix from Pol et al., (2012) [17] with Sahitisuchus fluminensis added. (JPG) [file pone.0081386.s002.jpg]
