# Supplementary material for: A New Sebecid from the Paleogene of Brazil and the Crocodyliform Radiation after the K–Pg Boundary
Source: PLoS One. 2014 Jan 15;9(1):e81386. doi: 10.1371/journal.pone.0081386 (PMC3893294; doi:10.1371/journal.pone.0081386)
Supplement: Data S1 — Phylogenetic Analyses. (DOC) [file pone.0081386.s003.doc]

**Supporting Information**

**Phylogenetic Analyses**

Main information concerning to the topologies of the resulting cladograms (**figure 8 and supporting figures S1** and S**2**).

Tree Length (**L**)= 1264 (unordered); 1324 (ordered).

Most Parsimonious trees (**MPTs**) = 5940 (unordered); 8640 (ordered).

Unambiguous characters states supporting some nodes.

**Sebecosuchia** node

Character 103- 2

Character 155- 0

Character 159- 2

Character 193- 0

**Sebecidae** node: ((*Iberosuchus* + *Bergisuchus* ) + *Barinasuchus* + (*Lorosuchus* + (*Sebecus* + *Langstonia* + *Sahitisuchus* + (*Ayllusuchus* + *Bretesuchus*))))

all trees:

Character 108- 0 (unordered)

Character 139- 0

some trees:

Character 286- 1

Character 56- 1 (ordered)

Character 78- 0 (ordered)

Character 141- 0 (ordered)

Character 149- 0 (ordered)

Character 157- 0 (ordered)

Character 179- 1 (ordered)

Character 186- 0(ordered)

Character 192- 0 (ordered)

Character 212- 1 (ordered)

Character 316- 1 (ordered)

Character 331- 1 (ordered)

Character 332- 1 (ordered)

Character 335- 1 (ordered)

node: (*Iberosuchus + Bergisuchus*)

Character 67- 1

node: (*Barinasuchus* + (*Lorosuchus* + (*Sebecus* + *Langstonia* + *Zulmasucchus* + *Sahitisuchus* + (*Ayllusuchus* + *Bretesuchus*))))

Character 121- 0

Character 226- 0

some trees:

Character 67- 2 (ordered)

node: (*Lorosuchus* + (*Sebecus* + *Langstonia* + *Zulmasuchus* + *Sahitisuchus* + (*Ayllusuchus* + *Bretesuchus*)))

all trees:

Character 42- 1

Character 292- 0

some trees (ordered):

Character 9- 1

node: (*Sebecus* + *Langstonia* + *Zulmasuchus* + *Sahitisuchus* + (*Ayllusuchus* + *Bretesuchus*))

all trees:

Character 155- 1

Character 219- 1

Character 220- 1

Character 288- 1

some trees:

Character 167- 1

Character 189- 1

node: (*Ayllusuchus* + *Bretesuchus*)

Character 226- 2

Character 285- 2
